# A Therapeutic Uricase with Reduced Immunogenicity Risk and Improved Development Properties

**DOI:** 10.1371/journal.pone.0167935

**Published:** 2016-12-21

**Authors:** Andrew C. Nyborg, Chris Ward, Anna Zacco, Benoy Chacko, Luba Grinberg, James C. Geoghegan, Ryan Bean, Michaela Wendeler, Frank Bartnik, Ellen O’Connor, Flaviu Gruia, Vidyashankara Iyer, Hui Feng, Varnika Roy, Mark Berge, Jeffrey N. Miner, David M. Wilson, Dongmei Zhou, Simone Nicholson, Clynn Wilker, Chi Y. Wu, Susan Wilson, Lutz Jermutus, Herren Wu, David A. Owen, Jane Osbourn, Steven Coats, Manuel Baca

**Affiliations:** 1 MedImmune LLC, Gaithersburg, Maryland, United States of America; 2 Ardea Biosciences, San Diego, California, United States of America; 3 MedImmune LLC, Mountain View, California, United States of America; 4 MedImmune LTD, Cambridge, United Kingdom; Tecnologico de Monterrey, MEXICO

## Abstract

Humans and higher primates are unique in that they lack uricase, the enzyme capable of oxidizing uric acid. As a consequence of this enzyme deficiency, humans have high serum uric acid levels. In some people, uric acid levels rise above the solubility limit resulting in crystallization in joints, acute inflammation in response to those crystals causes severe pain; a condition known as gout. Treatment for severe gout includes injection of non-human uricase to reduce serum uric acid levels. Krystexxa^®^ is a hyper-PEGylated pig-baboon chimeric uricase indicated for chronic refractory gout that induces an immunogenic response in 91% of treated patients, including infusion reactions (26%) and anaphylaxis (6.5%). These properties limit its use and effectiveness. An innovative approach has been used to develop a therapeutic uricase with improved properties such as: soluble expression, neutral pH solubility, high *E*. *coli* expression level, thermal stability, and excellent activity. More than 200 diverse uricase sequences were aligned to guide protein engineering and reduce putative sequence liabilities. A single uricase lead candidate was identified, which showed low potential for immunogenicity in >200 human donor samples selected to represent diverse HLA haplotypes. Cysteines were engineered into the lead sequence for site specific PEGylation and studies demonstrated >95% PEGylation efficiency. PEGylated uricase retains enzymatic activity *in vitro* at neutral pH, in human serum and *in vivo* (rats and canines) and has an extended half-life. In canines, an 85% reduction in serum uric acid levels was observed with a single subcutaneous injection. This PEGylated, non-immunogenic uricase has the potential to provide meaningful benefits to patients with gout.

## Introduction

Urate oxidase (uricase) is a homotetrameric enzyme composed of four identical 34 kDa subunits [1]. This enzyme initiates a series of reactions that convert uric acid (UA) to a more soluble and easily excreted product, allantoin. In short, uricase catalyzes the reaction of UA with O_2_ and H_2_O to form 5-hydroxy-isourate (HIU) and the release of H_2_O_2_ [2]. HIU is an unstable product that undergoes non-enzymatic hydrolysis to 2-oxo-4-hydroxy-4-carboxy-*5*-ureidoimidazoline (OHCU) which then decarboxylates spontaneously to form racemic allantoin [2, 3]. Living species that contain a functional uricase also express two additional enzymes (HIU hydrolase and OHCU decarboxylase) which catalyze these reactions more quickly to generate (S)-allantoin [2]. A functional uricase can be found in a wide range of organisms, such as: archaea, bacteria, and eukaryotes. However, in humans and some primates a functional uricase enzyme is not expressed [4, 5]. The lack of functional uricase expression in humans is due to three mutations that result in complete silencing of the gene [4]. A number of hypotheses have been proposed to explain the evolutionary elimination of uricase activity and commensurate increase in UA levels [6]. These include the idea that an increase in UA levels (powerful antioxidant and scavenger of oxygen radical) led to a decrease in oxygen free radical associated disease (cancer) and an increase in lifespan [7, 8]. Additionally, the fact that UA structurally resembles neuro-stimulants such as caffeine and theobromine has led to the speculation that increased UA levels may have led to an intellectual/cognitive jump however, this is controversial [9, 10]. It has also been suggested that an increase in UA led to and helped maintain blood pressure levels required by hominids while consuming a very low salt vegetarian diet [11, 12]. Lastly, the loss of uricase may have aided the accumulation of fat stores in response to fructose, a major nutrient in fruits that were a primary staple of ancestral simians [7, 13]. This would have been highly advantageous to frugivorous primates in a resource-constrained environment [4]. The potential benefits of increased serum UA levels for our predecessors notwithstanding, in modern humans, high uric acid may have negative consequences due to urate deposition, and an increase in gout.

Gout affects more than 8 million Americans and is an inflammatory arthritis defined as serum UA levels exceeding UA solubility limits in body fluids [14, 15]. Serum UA levels higher than 6.8 mg/dL can result in UA crystal formation in tissues, provoking an acute inflammatory response. Acute gouty arthritic attacks (flares) and chronic inflammation resulting from UA crystal deposits in fibrous tissues are painful and debilitating. The damage caused by gout can result in chronic pain, functional impairment, and compromised health-related quality of life. A variety of therapeutic agents exist for controlling hyperuricemia targeting either production or excretion of UA. Inhibitors of xanthine oxidase (enzyme that converts xanthine and hypoxanthine to UA) target the production side and have been prescribed since the 1960s [16]. The most common of these, Allopurinol, is used by more the 2 million gout patients in the US. However, many patients continue to have higher than acceptable UA levels despite this treatment [17]. Studies have shown that UA levels in patients can also be controlled by inhibiting URAT1, a renal urate-anion transporter responsible for UA reabsorption in the kidney. URAT1 inhibitors act in the proximal tubules in the kidneys, where they interfere with the URAT1-mediated absorption of UA from the kidney back into the blood thereby increasing excretion of UA [18]. URAT1 inhibitors, such as benzbromarone, probenecid and lesinurad, promote excretion of UA. Lastly, it has also been shown that treatment with exogenous uricase rapidly reduces UA levels in the peripheral blood stream by oxidizing UA to a more soluble product, allantoin. There are two clinically approved uricases, Krystexxa^®^ (pegloticase), which is approved for the treatment of Chronic Refractory Gout [19], and Elitek^®^ (rasburicase), which is approved for tumor lysis syndrome [20].

Krystexxa^®^ is a chimeric protein comprised of the pig and baboon uricase sequence and is hyper-PEGylated (~440 kDa PEG per tetramer) via random lysine conjugation to reduce immunogenicity and extend half-life [21]. It was first tested in Phase 1 clinical trials via a subcutaneous route of administration [22]. In that study an immune response was observed in some patients and antibodies to PEG were reported. Additional studies were performed using an intravenous (IV) route of administration [23]. Clinically approved Krystexxa^®^ is administered by an IV infusion over a 2 hour period. During phase 3 clinical trials, 26% of patients experienced infusion reactions and 6.5% of patients had reactions characterized as anaphylaxis. As a result, Krystexxa^®^ contains a black box warning for anaphylaxis and infusion reactions [19]. Patients are typically pretreated with antihistamines or corticosteroids prior to the IV infusion and then monitored post-infusion [24]. Pretreatment, IV-infusion and post-infusion monitoring takes 6–8 hours once every two weeks, a significant patient burden. In phase 3 clinical trials, a high percentage of patients developed anti-drug antibodies (~92%) with a strong antibody response to the PEG moiety of the molecule [19, 25]. Approximately 40% of patients experienced a positive primary endpoint (reduction in UA levels below 6 mg/dl for 6 months) [19]. In spite of the infusion reactions, anti-drug response, and inconvenient dosing schedule, dramatic results have been observed in clinical trials and case studies demonstrating the reduction or resolution of tophi (UA crystal deposits). Digital photos of patients with tophaceous gout (hands or feet) before and after multiple Krystexxa^®^ treatments have demonstrated the potential for an uricase in resolving tophi and UA burden [21]. Elitek^®^ (rasburicase) is also a marketed uricase which has a black-box warning for anaphylaxis and hemolysis (especially in patients with a G6PD deficiency) [26]. Rasburicase is a modified recombinant *Aspergillus flavus* uricase [27]. It has a half-life of 16–21 hours in humans and must be dosed daily via IV infusion [28]. The protein is potentially immunogenic and anaphylactic. Dosing frequency (daily), route of administration (IV), immunogenicity, and cost make rasburicase an unlikely option for chronic gout treatment. However, it is approved for acute and rapid increases in UA due to chemotherapeutic tumor lysis (tumor lysis syndrome) which can lead to complications such as a renal failure.

Herein we describe the development of a therapeutic uricase with the following characteristics: 1) soluble expression and activity at neutral pH; 2) site specific PEGylation in order to minimize the amount of PEG needed to extend half-life and provide drug product homogeneity, thereby lowering potential PEG immunogenicity; 3) low immunogenic potential as measured in a T-cell immunogenicity assay; 4) excellent *in vivo* activity (whole blood UA reduction) and half-life and 5) potential for subcutaneous route of administration. Screening and analysis has led to the selection of a lead uricase from hundreds of potential uricase species and candidate sequences. The lead uricase (*Arthrobacter globiformis*) expresses at high levels in a soluble form in *E*. *coli*, has been engineered to reduce sequence liabilities, is highly active and stable at neutral pH, in human serum, *in vivo* and contains two engineered Cys residues per monomer for site-specific PEG conjugation. The lead molecule reduced serum UA in canines by ~85% with a single subcutaneous (SC) injection. In addition, the lead uricase has been screened against >200 human normal donor peripheral blood mononuclear cell (PBMC) samples for T-cell immunogenicity and demonstrated low potential for immunogenicity.

## Results

### Selection of Uricase

Mammalian expressed uricases are localized to an intracellular high pH peroxisome compartment. Many of these uricases are insoluble at neutral pH which presents a challenge for recombinant production and characterization [29, 30]. We attempted to identify uricases that might have favorable properties for manufacturing and activity in the peripheral blood stream (neutral pH, 37°C). Candidate uricases were selected using criteria that included (but not limited to): favorable biological properties (such as soluble expression in *E*. *coli*, neutral pH solubility, and activity), low sequence identity or similarity to other selected sequences (diversity), low endogenous Cys content, and organisms with a uricase that might have favorable biochemical properties (extremophile, thermophile, acidophile, etc). The following seven candidate microbial sequences were chosen for further investigation (S1 Fig): *Arthrobacter globiformis* uricase (NCBI Accession number D0VWQ1), *Deinococcus geothermalis* uricase (NCBI Accession number WP_011525965), *Deinococcus radiodurans* uricase (NCBI Accession number WP_010887803), *Granulicella tundricola* uricase (NCBI Reference Sequence: WP_013581210.1), *Solibacter usitatus* uricase (NCBI Accession number WP_011682147), *Terriglobus saanensis* uricase (NCBI Accession number WP_013569963) and *Kyrpidia tusciae* uricase (NCBI Accession number ADG06709). Additionally, a synthetic uricase was generated from the consensus uricase sequence (S1 Fig) derived from the alignment of 50 uricase sequences that shared the greatest identity to *Arthrobacter globiformis* uricase sequence (49–79% sequence identity). Uricases representing broad species diversity were included (animal, plant, microbial). As shown in Table 1, there is a significant amount of sequence diversity across the 8 sequences.

**Table 1:** Uricase sequence identity comparison.

	Consensus	*Arthrobacter globiformis*	*Deinococcus geothermalis*		*Deinococcus radiodurans*	*Granulicella tundricola*	*Solibacter usitatus*	*Terriglobus saanensis*	*Kyrpidia tusciae*
Consensus	100	61.7		44.4	44.1	36.7	28.0	39.4	28.5
	*Arthrobacter globiformis*	100		41.2	43.4	42.7	29.2	38.4	27.3
		*Deinococcus geothermalis*		100	75.1	36.0	23.4	34.5	22.2
				*Deinococcus radiodurans*	100	36.3	23.1	37.0	21.6
					*Granulicella tundricola*	100	26.4	58.4	27.1
						*Solibacter usitatus*	100	21.5	43.7
							*Terriglobus saanensis*	100	25.6
								*Kyrpidia tusciae*	100

### Screening Paradigm

**Fig 1:**
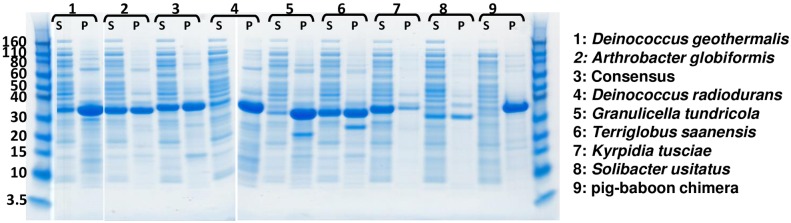
Expression of candidate uricase in *E*. *coli*. 8 uricase sequences were expressed in *E*. *coli*and evaluated for soluble (S) and insoluble (P) expression level. As shown in Fig 1, most uricases were present at high level in the insoluble (P) material. The pig-baboon chimera appears to express almost entirely in the insoluble (P) fraction (Lane 9). Cytosolic soluble (S) expression was considered favorable.

A screening paradigm was used to identify candidates for further optimization. The 8 uricase sequences described above were cloned with an amino terminal His tag and expressed in *E*. *coli*. Each uricase construct was evaluated for expression level and in particular, soluble expression at 37°C. Uricase expressing *E*. *coli* were lysed and soluble material was separated from insoluble (pellet) material. As shown by SDS-PAGE analysis in Fig 1, under the expression conditions used, most uricases were present at high level in the pellet (P) material but not in the soluble (S) fraction. A pig-baboon chimera representing the uricase sequence in Krystexxa^®^ was also generated but appeared to partition entirely in the insoluble fraction and was not evaluated further (Fig 1, Lane 9). Cytosolic soluble expression was considered to be a favorable property. The 8 uricases were then purified from the soluble fractions of *E*. *coli* cell lysates by Ni-affinity chromatography. Protein yield was determined by measuring the absorbance at 280 nm, and measurement of intact mass was performed by mass spectrometry (Table 2). Uricase enzymatic activity requires that the enzyme form a homo-tetramer. Homo-tetramer formation was confirmed by size exclusion chromatography coupled with light-scattering detection (Table 2). Three uricases were eliminated from further evaluation based on unfavorable expression, solubility or purification yields, namely; *Solibacter usitatus*, *Kyrpidia tusciae*, and *Granulicella tundricola* uricases.

**Table 2:** Mass Spec and SEC-LS Analysis of candidate uricases.

	Predicted Mass (kDa)	Measure Mass Monomer (kDa)	Theoretical Tetramer (kDa)	Measured SEC-LS Tetramer (kDa)	Tetramer Formation
*Arthrobacter globiformis*	33.88	33.88	135.52	135.20	**✓**
*Deinococcus geothermalis*	35.19	35.19	140.76	136.30	**✓**
*Terriglobus saanensis*	32.69	32.69	130.76	126.80	**✓**
Consensus	35.83	35.83	143.32	141.30	**✓**
*Deinococcus radiodurans*	35.58	35.58	142.32	140.40	**✓**
*Granulicella tundricola*	33.60	33.60	134.40	128.00	**✓**
*Kyrpidia tusciae*	38.24	38.24	152.96	147.20	**✓**
*Solibacter usitatus*	33.24	33.24	132.96	137.70	**✓**

**Fig 2:**
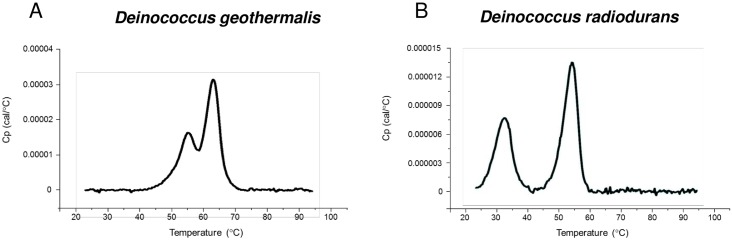
Differential scanning calorimetry measurements. Thermal stability was assessed for all candidate usicases by differential scanning calorimetry. Two transitions were observed for each uricase (TM1 and TM2). *Deinococcus geothermalis* uricase (A) has a low TM1 (32°C) whereas *Deinococcus radiodurans* uricase (B) has a TM1 of 55°C suggesting that *Deinococcus geothermalis* uricase is more thermally stable.

Differential scanning calorimetry measurements were performed to assess thermal stability. Consistent with data reported previously (*Aspergillus flavus* uricase) [31], two thermal transitions (TM1 and TM2) were observed for each uricase. *Terriglobus saanensis* and *Deinococcus radiodurans* uricases exhibited a thermal transition (TM1) that was lower than desired and, as a result, these two uricases were eliminated from the pool of candidates (Table 3). Fig 2 shows two examples of the differential scanning calorimetry results (*Deinococcus geothermalis* and *Deinococcus radiodurans* uricases).

**Table 3:** Differential scanning calorimetry stability comparison.

	TM1 (°C)	TM2 (°C)
*Arthrobacter globiformis*	47.5	73.0
*Deinococcus geothermalis*	55.0	63.0
*Terriglobus saanensis*	42.0	90.0
Consensus	56.0	69.0
*Deinococcus radiodurans*	32.0	54.0

**Fig 3:**
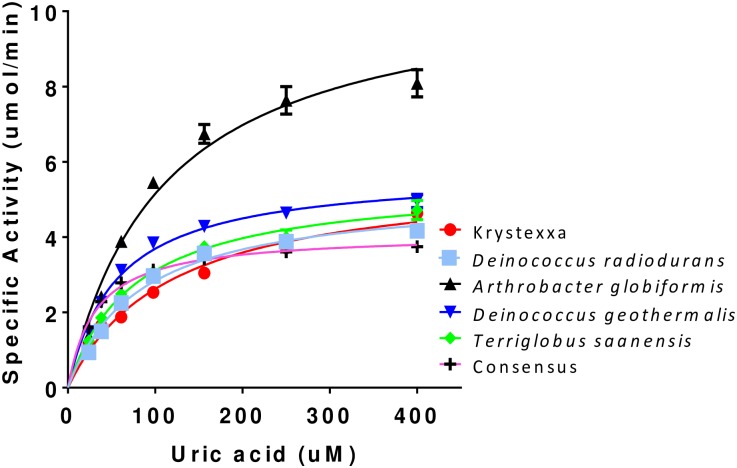
Enzymatic activity comparison. Substrate (UA) depletion assays were performed to assess uricase activity. The rate (specific activity) of UA oxidation was calculated based on a linear decrease in absorbance. Solid lines represent Michaelis-Menten kinetic fits performed in Prism GraphPad. Relative to Krystexxa^®^, *Arthrobacter globiformis* uricase had a 2 fold better kcat and *Deinococcus geothermalis* uricase had a 2 fold better K_M_ (Table 4).

Five uricases from *Arthrobacter globiformis*, *Deinococcus geothermalis*, *Deinococcus radiodurans*, *Terriglobus saanensis* and the consensus uricase were evaluated for catalytic activity. Nearly all uricases are active at pH >8.0 [32] and most uricase activity assays reported in the literature are performed at alkaline pH between 8.0 and 9.0. However, a therapeutic uricase needs to be stable and active in the peripheral blood stream at neutral pH. As a result, activity assays were performed at pH 7.4. The substrate depletion assay was amenable to kinetic monitoring of UA oxidation at a variety of substrate concentrations. From these data, kcat and K_M_ were calculated for each uricase (Table 4). Substrate concentration versus specific activity curves and Michaelis Menten fits are shown in Fig 3.

**Table 4:** Enzymatic activity comparison (kcat and K_M_).

	kcat (uM UA/S/uM uricase)	K_M_ (uM UA)
Krystexxa^®^	3.20	116.3
*Arthrobacter globiformis*	6.08	109.7
*Deinococcus geothermalis*	3.24	55.73
*Terriglobus saanensis*	3.09	76.18
Consensus	2.30	31.76
*Deinococcus radiodurans*	2.93	83.06

Based on all screening criteria, two uricases emerged with the most favorable properties (expression, solubility, thermal stability, native Cys content, etc). Each of these had somewhat different kinetic parameters. *Arthrobacter globiformis* uricase had an improved kcat and *Deinococcus geothermalis* uricase had an improved K_M_ relative to Krystexxa^®^. Gout and tumor lysis syndrome patients typically have saturating levels of UA (>6.8 mg/dl, 408 μM). Therefore, we reasoned that kcat may be the more relevant kinetic parameter for a therapeutic uricase. Dose models were generated based on an improvement in kcat (*Arthrobacter globiformis)* or K_M_ (*Deinococcus geothermalis*) relative to Krystexxa^®^. The modeling predicted an improved K_M_ (*Deinococcus geothermalis*) would provide no advantage in dosing amount or frequency. However, an improved kcat (*Arthrobacter globiformis*) was predicted to provide an advantage in terms of reduced dosing amount and/or frequency. Thus, the results of the dose modeling confirmed that kcat is the more relevant catalytic parameter for a therapeutic uricase. Based on the screening criteria and the improved kcat, *Arthrobacter globiformis* uricase was selected for further characterization and optimization.

### Sequence Liabilities and Modifications

**Fig 4:**
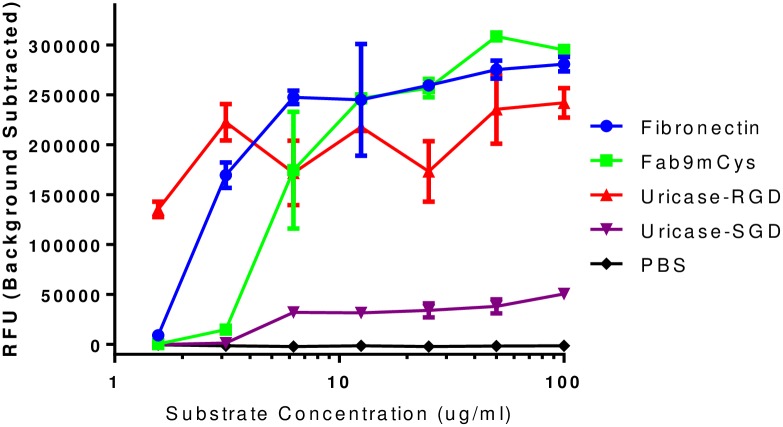
Integrin binding analysis. The tripeptide, RGD has been shown to mediate cell adhesion through integrin binding. An M21 tumor cell adhesion assay was conducted to determine if the RGD motif in *Arthrobacter globiformis* uricase is surface accessible. M21 cells were used because they express α_v_β_3_ and α_v_β_5_ integrins. RGD-containing fibronectin substrate (positive control), Fab9mCys (an IgG that contains an RGD within the CDR-H3 loop), and uricase containing RGD all bind the M21 cells as measured by increase in fluorescence. However, PBS (negative control), and uricase containing the SGD had limited increase in fluorescence.

The *Arthrobacter globiformis* uricase sequence contains an Arginine-Glycine-Aspartate (RGD) motif (amino acids 49, 50 and 51 in the native sequence). The tripeptide RGD within fibronectin has been shown to mediate cell adhesion by binding to integrins which are a major family of cell adhesion receptors known to play a key role in cell-cell and cell-extracellular matrix interactions [33]. Integrin binding therapeutics have been explored in a number of disease areas including oncology, infection, and thrombosis [34]. Putatively, an RGD integrin binding motif could be problematic for a therapeutic protein that needs to function in the peripheral blood stream. An M21 tumor cell adhesion assay was conducted to determine if the *Arthrobacter globiformis* uricase RGD was capable of interacting with α_v_β_3_ and α_v_β_5_ integrins which are expressed on the surface of M21 cells [35]. Results are shown in Fig 4. These data indicate that the RGD-containing *Arthrobacter globiformis* uricase can bind M21 cells which may be predictive of the potential for undesired integrin binding. A peptide blocking assay was performed to verify that the RGD-containing uricase was binding to M21 cells via the RGD motif. In these assays a soluble control RGD or RGE containing peptide was used to compete for binding. Only the RGD-containing peptide competed for binding suggesting that M21 cell binding was integrin-mediated via the RGD binding motif (S2 Fig).

**Fig 5:**
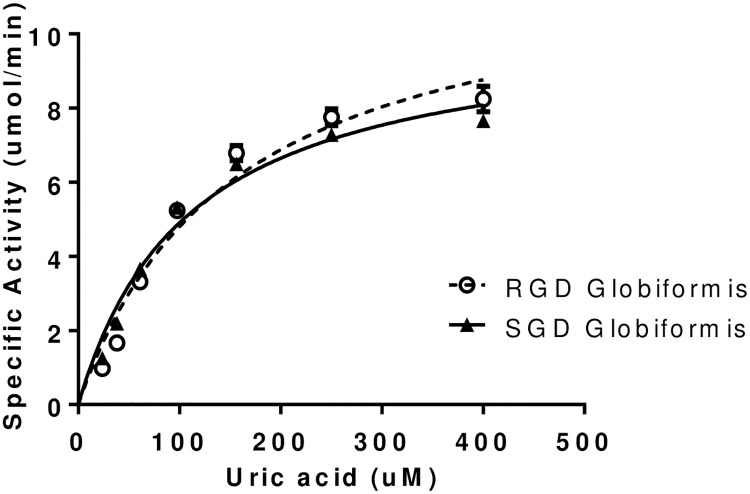
Enzymatic activity comparison of RGD and SGD containing uricases. Substrate (UA) depletion assays were performed to assess uricase activity. The rate (specific activity) of UA oxidation was calculated based on a linear decrease in absorbance. Solid lines represent Michaelis-Menten kinetic fits performed in Prism GraphPad. Both RGD and SGD containing *Arthrobacter globiformis* uricases appear to have comparable enzymatic activity.

Although it is unknown if an RGD motif in a therapeutic protein is problematic, we opted to remove this potential liability. Based on the alignment of 50 uricase sequences that are most closely related to the *Arthrobacter globiformis* uricase sequence, we noted that Glycine (G) and Aspartate (D) in the RGD motif were highly conserved across the alignment set. The consensus residues for these positions were G and D. However, the Arginine (R) was less conserved and the consensus residue at this position was a Serine (S). Therefore, site-directed mutagenesis was used to replace the R in the RGD motif with an S, thus making the RGD an SGD (R49S). The R49S mutation (SGD) led to a marked reduction in binding to M21 cells (Fig 4). The R49S variant (SGD) uricase was evaluated for activity at pH 7.4 and was found to be equivalent to the wild type enzyme (Fig 5).

### Protein Immunogenicity Analysis (Epibase^®^)

To assess the potential immunogenic risk in humans, the engineered *Arthrobacter globiformis* uricase protein was screened in the Epibase^®^ immunogenicity assay. The LONZA Epibase^®^ assay is a human (PBMC based T-cell immunogenicity assay used to assess “immunogenicity risk”. In the format used, test proteins were incubated with donor PBMC samples during which natural cellular uptake and proteolytic processing must occur in order for a peptide to bind to MHC and elicit a CD4^+^ T-cell response. Although this assay cannot necessarily predict clinical immunogenicity, it can be used to identify “high risk” and “low risk” proteins based on the number of responder PBMC samples and the overall response magnitude (Stimulation index). PBMC samples from 202 normal donors were used to screen the T-cell immunogenicity of the uricase candidate relative to a negative control (buffer) and a positive control protein (KLH). The 202 donors were selected to represent HLA-DRB1 frequencies in the Caucasian population (S3 Fig).

**Fig 6:**
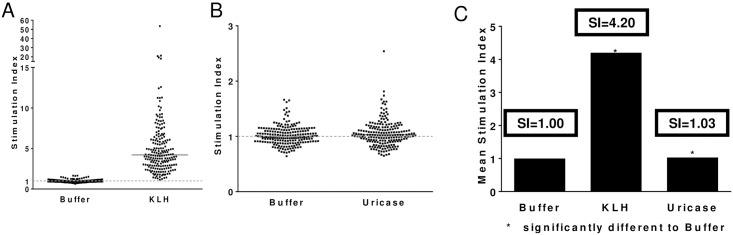
Ex vivo immunogenicity analysis. The Epibase^®^ assay is a human PBMC T-cell immunogenicity assay used to assess “immunogenicity risk”. In this assay, PBMC samples from 202 normal donors were used to screen the T-cell immunogenicity of a modified *Arthrobacter globiformis* uricase candidate relative to a negative control (buffer) and a positive control (KLH). The 202 donors were selected to represent HLA-DRB1 frequencies observed in a Caucasian population (S4 Fig). Stimulation indices (SI) values describe the ratio of proliferating CD3^+^CD4^+^ T-cell in antigen treated versus untreated wells. SI values >2 are considered positive which is supported by a p-value <0.05. A) Shows the individual SI values for negative control—0/202 donor samples (0%) responded with SI>2.0; and positive control—181/202 donors (91%) responded with a SI>2.0. B) Shows the individual SI values for negative control and modified uricase candidate—1/202 donor samples responded with a SI>2.0. C) Shows the mean stimulation index for all donor samples for buffer (SI = 1.0), KLH SI = 4.2 and modified *Arthrobacter globiformis* (SI = 1.03).

Positive and negative controls behaved as expected: negative control—0/202 donor samples (0%) responded with a mean population SI = 1.0 and positive control—181/202 donors (91%) responded with a mean SI = 4.2. Surprisingly, only one donor sample responded to the *Arthrobacter globiformis* uricase candidate—1/202 donor samples (0.5%) with a mean population SI = 1.03; Individual donor data are shown in Fig 6A–6C. Fig 6A shows that the buffer (negative control) stimulation index is 1.0 and the KLH (positive control) had a 91% response (SI>2). The KLH mean total SI = 4.2. Fig 6B shows the donor response from *Arthrobacter globiformis* uricase candidate in comparison to the buffer control. Only one donor responded to the *Arthrobacter globiformis* uricase sequence with an SI>2. An alternative method for analyzing the data is to look at the overall stimulation index for all donors and compare that to buffer and the positive control. Fig 6C shows that the mean buffer stimulation index is 1.0, KLH is 4.2, and the mean for *Arthrobacter globiformis* uricase SI is 1.03. Despite the microbial origin of this protein, these data would suggest that the human immunogenicity risk is low.

### Site Specific PEGylation

Polyethylene glycol (PEG) conjugation is routinely used to modify proteins in order to extend half-life [36]. Modification of proteins with PEG can be performed as either random attachment to selected protein residues (e.g. lysine side chains in the case of Krystexxa^®^), or site-specific to unique predetermined site(s). The latter approach has the advantage that the conjugation chemistry can be better controlled and manufactured consistently, yielding a highly homogenous PEGylated product with defined bioactivity. Among methods for site-specific attachment, the most widely used approach is coupling to unpaired cysteine (Cys) residues. Sites for Cys introduction can be carefully selected to limit or avoid any negative impact on bioactivity or biophysical properties of the conjugate product following modification with PEG. Given the absence of any Cys residues within the engineered *Arthrobacter globiformis* uricase sequence, modification of this protein with thiol-reactive reagents would be confined to sites where Cys residues have been introduced (described below). Sites for Cys residue introduction in the *Arthrobacter globiformis* uricase sequence were selected after the following criteria were taken into consideration

i Sites must be on a solvent-exposed surface of the protein to ensure efficient reaction with a thio-reactive PEG reagent;
ii Sites must not be close to the enzyme active sites to avoid the risk of impacting activity; and
iii Sites must not be in close proximity to each other so that PEGylation of one site does not sterically hinder PEGylation of other sites.

Given the tetrameric nature of uricase, intra- and inter-subunit distances were both considered in the cases of ii. and iii.

In order to compute parameters relevant to these considerations, the three-dimensional structure of the *Arthrobacter globiformis* uricase bound to UA was used (PDB accession code: 2YZB) [1]. The atomic coordinates for this structure were used to compute the following set of parameters:

i Solvent accessible surface area for each amino acid residue within the structure
ii Atomic distances between each side chain Cα atom and the C5 atom of the UA substrate (Cα-C5 distance).

To identify preferred positions within the *Arthrobacter globiformis* uricase for substitution with Cys, the following criteria were initially set. First, residues were identified with total solvent accessible surface area > 100Å^2^ and a Cα-C5 distance > 25Å (i.e. to each C5 in the 4 UA molecules bound to the uricase tetramer). Second, as a further restriction, for any given uricase residue, these criteria had to be met in all four subunits. Of the 287 amino acid residues in each uricase subunit, only 9 satisfied these criteria. These were Thr11; Asn33; Asn119; Asp120; Ser142; Glu196; Pro238; Glu286 and Arg289. A third criterion was then considered by calculating the matrix of atomic distances between pairs of Cα atoms within this set of residues across the tetrameric structure (S1 Table). From this analysis, Thr11, Asn33, Glu196 and Asn119 were selected as preferred residues for substitution with Cys, as their Cα atoms across the tetramer are well separated (≥ 19.5 Å for all pairs).

### Cys Incorporation and PEGylation

**Fig 7:**
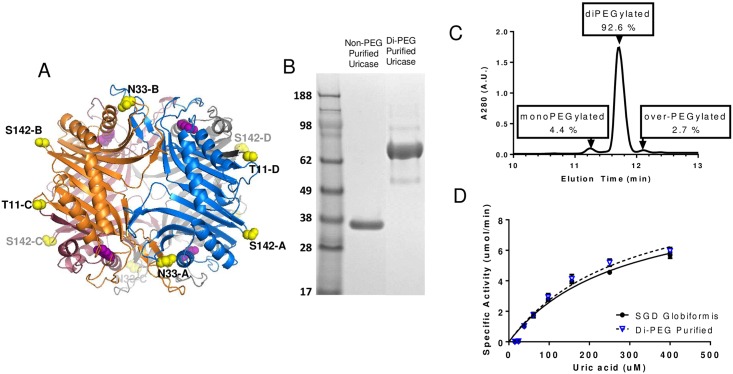
PEGylation strategy and analysis. (A) Shows the three dimensional solvent accessible sites within the tetrameric crystal structure of *Arthrobacter globiformis* uricase [1]. Each uricase monomer subunit of the tetrameric enzyme is shown in a different color, and residues selected for substitution with Cys (T11, N33, S142) are shown in yellow. (B) SDS-PAGE analysis of di-PEGylated and non-PEGylated uricase suggests that the di-PEGylated material runs at a much higher molecular weight relative to the non-PEGylated material. (C) A reverse-phase chromatography analysis of the di-PEGylated material suggests that 92.6% of the material is di-PEGylated, 4.4% mono-PEGylated, 0% unreacted and 2.7% over-PEGylated. (D) Demonstrates that the activity of di-PEGylated uricase is comparable to non-PEGylated uricase.

In order to incorporate enough PEG to extend *in vivo* half-life but minimize the total amount of PEG to reduce the potential for an immune response we incorporated 2 (T11C, N33C) or 3 (T11C, N33C, and S142C) Cys residues per modified *Arthrobacter globiformis* uricase subunit. Fig 7A shows the three dimensional solvent accessible sites within the tetrameric crystal structure of *Arthrobacter globiformis* uricase (PDB accession code: 2YZB) [1]. Each uricase monomer subunit of the tetrameric enzyme is shown in a different color, and residues selected for substitution with Cys (T11, N33, S142) are shown in yellow. These side chains are highly surface exposed, distant from each other, and distant from each active site within the tetramer. The two Cys containing variants (T11C, N33C and T11C, N33C, S142C) were analyzed for expression, solubility, purity, and activity both before and after PEGylation. Due to the solvent exposed side chains for the introduced Cys residues, these constructs tended to undergo disulfide-mediated aggregation unless they were kept under reducing conditions. This necessitated that a reducing agent (1–5 mM dithiothreitol) be present during purification and assay procedures, but once the Cys had been PEGylated, reducing agent was no longer necessary. Both Cys-containing constructs expressed at a similar level to the wild type uricase and demonstrated similar enzymatic activity (S5 Fig). All assays were run in the presence of dithiothreitol to eliminate the potential for disulfide bonding of the unmodified proteins.

### Analysis of PEGylated Uricase

Di and tri-PEGylated uricases were generated by modification of the unpaired Cys residues with a 10 kDa PEG reagent containing a thiol-reactive maleimide group. Analysis of di-PEGylated material by SDS-PAGE (Fig 7B) confirmed that most of the protein was uniformly conjugated with PEG. Reverse phase chromatography analysis of the di-PEGylated material suggested that 92.6% was di-PEGylated, 4.4% mono-PEGylated and a small amount of material over-PEGylated (~3%) was observed (Fig 7C). PEGylation did not appear to impact the enzymatic activity. Di-PEGylated material showed a similar rate of UA oxidation compared to the non-PEGylated enzyme (Fig 7D). Comparable results were obtained for the tri-PEGylated material (not shown). These methods of analysis disrupt the quaternary structure of the uricase, but as a homo-tetramer the predominant native state product for the di-PEG uricase would be expected to have 8 x 10 kDa PEG chains, while the tri-PEG uricase would be expected to have 12 x 10 kDa PEG chains.

### In vivo PK for Di and Tri-PEGylated Uricase

**Fig 8:**
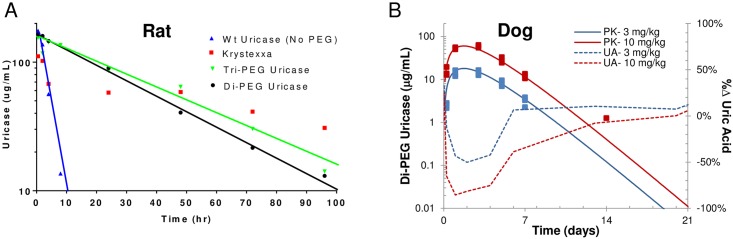
In vivo rat PK and dog PK/PD assessment. (A) *In vivo* rat PK comparison of non-PEGylated (blue triangles), di-PEGylated uricase (black circles) and tri-PEGylated uricase (green inverted triangles) and Krystexxa^®^ (red squares). Rats were dosed IV at 5 mg/kg and samples were collected various time points and analyzed for residual uricase activity above background. Representative data from individual rats are shown. Both di-PEGylated and tri-PEGylated uricases have mono-phasic profiles. The half-life for the di-PEGylated uricase was ~22.8 hours and tri-PEGylated ~29.9 hours. Non-PEGylated uricase half-life in rats was 2–3 hours. Krystexxa^®^ (red squares) had an atypical initial elimination profile followed by a relatively linear PK profile. (B) *In vivo* canine PK/PD study of di-PEGylated uricase delivered via SC route of administration at 3 mg/kg (blue squares) or 10 mg/kg (red squares). Hashed lines are associated with the right axis and represent the % UA measured in the blood.

The pharmacokinetic (PK) behavior of PEGylated and non-PEGylated uricases administered intravenously was studied in rats. A rat study was chosen because there was precedence for testing PK of PEGylated uricases in rats [37]. Rats were dosed IV at 5 mg/kg and serum samples were collected at various time points and analyzed for uricase activity in order to compute the concentration of active enzyme. The enzymatic specific activity of the uricase that went into the rats (predose) and the uricase that was measured from serum (postdose) was comparable suggesting activity was retained during the *in vivo* study. Fig 8A demonstrates that di and tri-PEGylated uricases have substantially longer half-lives than the non-PEGylated uricase. Non-PEGylated uricase had a half-life of 2–3 hours in this study (Fig 8A, blue triangles). Both di and tri-PEGylated uricases exhibit monophasic elimination profiles. The half-lives, volume of distribution (Vd) and clearance rate for each uricase are shown in Table 5. Di and tri-PEG showed very similar pharmacokinetic profiles with a reduction in clearance rate of tri-PEG over di-PEG. PEGylation resulted in a substantial improvement in *in vivo* half-life. We also evaluated the pharmacokinetic behavior of Krystexxa^®^ in the same rat PK study. Unlike the di and tri-PEGylated uricases, Krystexxa^®^ did not exhibit monophasic elimination, but a complex profile in which Krystexxa^®^ was rapidly eliminated in the first 2 hours followed by a more gradual elimination profile (Fig 8A, red squares). A linear fit was not included in Fig 8A for Krystexxa^®^ due to the elimination profile that is distinctly different from that of the di and tri-PEGylated uricases.

**Table 5:** In vivo rat PK comparison of di and tri-PEGylated uricases.

	Half-Life (hr)	Vd (L/kg)	Clearance (L/hr/kg)
Di-PEGylated	22.8 (7.4)	0.03 (7.2)	0.00096 (8.1)
Tri-PEGylated	29.9 (12.1)	0.03 (25)	0.00077 (12.8)

Coefficient of variation is expressed as a percentage within the parentheses

The PK of di-PEGylated uricase administered SC was studied in canines. A canine study was chosen because there was precedence for testing PK of PEGylated uricase in canines (Pegloticase/Krystexxa FDA BLA No. 125293, section 2.6.5.4.1). Canines were dosed SC at 3 and 10 mg/kg and serum or blood samples were collected at various time points and analyzed for uricase activity (PK) or uric aicd (PD). The enzymatic specific activity of the uricase that went into the canines (predose) and the uricase that was measured from serum (postdose) was comparable suggesting activity was retained during the *in vivo* study. Fig 8B demonstrates that di-PEGylated uricase delivered to canines via SC route of administration had a half-life of 1.81 ±0.31 days for 3 mg/kg (n = 3) and 1.82 ±0.22 days for 10 mg/kg (n = 3). A substantial reduction (~85%) in UA levels was observed and appears to be proportional to serum uricase levels (Fig 8B). Blood UA levels returned to normal as the uricase levels were depleted.

### Ex vivo Evaluation of Activity and Stability

**Fig 9:**
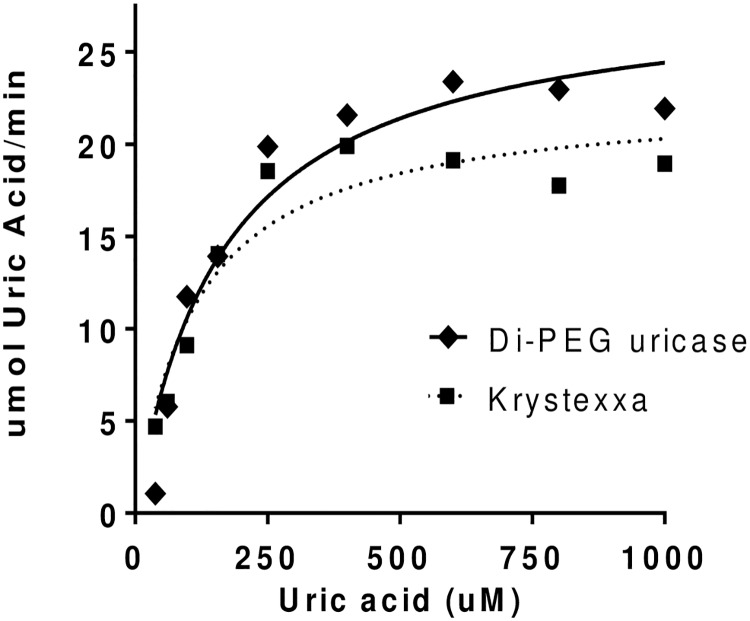
Enzymatic activity analysis in human serum. Substrate (UA) depletion assays were performed to assess uricase activity in 50% human serum. The rate (specific activity) of UA oxidation was calculated based on a linear decrease in absorbance. Solid (or dotted) lines represent Michaelis-Menten kinetic fits performed in Prism GraphPad. Di-PEGylated uricase activity was measured relative to Krystexxa^®^.

Di-PEG uricase activity was evaluated in 50% human serum (whole blood contains ~50% serum) at 37°C to mimic the complex *in vivo* matrix environment and temperature. This assay was performed similarly to other activity assays; however, UA absorbance could not be continually monitored at 292 nm due to the background absorbance due to human serum. As a result, assays were quenched at various time points using 50% percholoric acid which also precipitates protein material but not UA [38, 39]. Absorbance was then measured at 292 nm free of interfering species. Fig 9 shows comparable activity of di-PEGylated uricase and Krystexxa^®^ in 50% human serum at 37°C. A human serum based stability assay was also performed. Di-PEGylated uricase was incubated in 50% human serum for 0 to 24 hours at 37°C and then assayed for activity (data not shown). Di-PEG uricase retained activity following incubation in human serum. These data complement those from the rat PK study and suggest that di-PEGylated uricase is stable at 37°C in whole blood and serum.

## Discussion

The following criteria were used as a guide to develop a uricase with improved properties: 1) high level *E*. *coli* expression and good solubility at neutral pH; 2) site specific PEGylation in order to extend half-life, provide a homogenous drug product and thereby minimize the potential PEG immunogenicity; 3) low risk of immunogenicity and 4) potential for a convenient subcutaneous route of administration. The mammalian-derived uricase used to generate, Krystexxa^®^ (pegloticase) is a pig-baboon chimeric sequence that is isolated from *E*. *coli* in an insoluble form, and requires solubilization at high pH prior to PEGylation [40]. The protein is PEGylated via random lysine conjugation to achieve neutral pH solubility. Treating patients with Krystexxa^®^ is burdensome requiring pretreatment with antihistamines and corticosteroids (to prevent infusion reactions) and then an IV infusion which takes several hours in an infusion center. The Krystexxa^®^ label contains a black box warning for infusion reactions and anaphylaxis [19]. As stated previously, Krystexxa^®^ has a high rate of clinical immunogenicity [19]. The immunogenicity (antibody response) associated with Krystexxa^®^ has been shown to be primarily directed against the PEG moiety [25]. Immunogenicity to PEG has not been seen for most PEGylated therapeutics. As a result, the mechanism by which the human immune system mounts an anti-PEG response to a PEGylated protein is not well understood and somewhat controversial in the literature [25, 41, 42]. The Krystexxa^®^ tetramer is ~134 kDa and it has been reported to contain ~440 kDa of PEG per active tetramer (i.e. 11 x 10 kDa PEG per uricase subunit) [21], making the drug substance about 574 kDa in size. It has been hypothesized that the excessive amount of PEG conjugate along with the lack of homogeneity (random lysine conjugation) is responsible for the anti-PEG immune response that facilitates blood clearance [43]. Indeed, given the atypical elimination profile of Krystexxa^®^ following intravenous administration to rats (Fig 8) an early immune clearance mechanism of action may be associated with Krystexxa^®^ as suggested by others [43].

We initially identified an uricase that was highly expressed in *E*. *coli*, soluble at neutral pH, thermally stable (differential scanning calorimetry) and in short, contained favorable developability properties. The protein has been engineered to reduce putative therapeutic sequence liabilities like an RGD motif and unpaired C-terminal Cys. In addition, we have used protein structure modeling to identify optimal sites for incorporation of Cys residues for site specific PEG conjugation. These sites were selected based on their surface exposure, distance from the active site, and intramolecular distance from each other. Out of four potential sites that met all of the criteria, three were selected and uricase variants containing 2 or 3 exposed Cys residues per subunit were generated. These variants were PEGylated (10 kDa PEG) and analyzed *in vitro* and *in vivo*. In order to maintain developability and PK criteria, our lead uricase, as an active tetramer, contains 70–80 kDa of PEG (2 PEG per uricase monomer). This has the potential to reduce or avoid anti-PEG immunogenicity relative to Krystexxa^®^ which contains 400–440 kDa per active uricase tetramer. The lead di-PEGylated uricase retained activity in rat, dog (*in vivo*), and in human serum (*ex vivo*). Half-life of the di-PEGylated uricase was substantially extended to 22.8 hours in rats as opposed to ~2 hours for the non-PEGylated. A SC route of administration of the di-PEGylated uricase in canines led to a substantial reduction (~85%) in blood UA which was proportional to the uricase level as measured by an activity assay. After administration, uric acid levels dropped rapidly. These low levels were sustained for at least 72 hours, returning to baseline by Day 7. Significant UA lowering was seen in canines with two different doses of di-PEGylated uricase and both exhibited a similar time course of suppression. This level of UA decrease suggests that this molecule would be extremely effective at lowering uric acid humans.

Due to the fact that uricase is completely foreign to the human immune system and our uricase is microbial in origin, we screened the engineered *Arthrobacter globiformis* uricase protein for T-cell immunogenicity in 202 PBMC donor samples selected to represent a cross section of HLA-DRB1 frequencies. Surprisingly, in these assays only 1 donor sample responded to the protein with a SI > 2 and the total increase in the SI index score for the entire population went from 1.00 (buffer) to 1.03 (uricase). Although these assays cannot predict clinical immunogenicity, they have been used to predict immunogenicity risk associated with therapeutic biologics [44, 45]. Our data suggest that the protein is “low risk” when it comes to clinical immunogenicity, but this remains to be tested.

Based on the *in vivo* PK, activity, and preliminary formulation studies, our human dose projections suggest that a convenient route of administration is achievable. The uricase described herein has the potential to provide a safe and efficacious alternative urate lowering therapy to patients with severe gout or high urate burden (tumor lysis syndrome). Animal models are not predictive of clinical immunogenicity so human clinical trials will be required to further understand immunogenicity, dose level and frequency. The improved properties of this novel uricase warrant clinical studies in patients with severe gout or tumor lysis syndrome [46].

## Materials and Methods

### Protein Expression and Purification

Synthetic gene fragments encoding each uricase were obtained from GeneArt and cloned into a modified pET15b-based bacterial expression vector (Novagen) downstream of an N-terminal His-tag sequence (see protein sequences shown in S1 Fig). Sequences of all constructs were confirmed by DNA sequencing at Genewiz in Frederick, MD. Chemically competent *E*. *coli* BL21 (DE3, Lucigen) were transformed with the constructs described above and protein was produced by overnight growth in auto-inducing Magic Media (Invitrogen) at either 37 or 30°C. Cells were harvested by centrifugation and the pellet was frozen to assist in cell lysis. After thawing, pellets were resuspended in lysis buffer (10 mM sodium phosphate pH 7.2, 300 mM NaCl, 10 mM imidazole, 0.5 mg/mL lysozyme and 0.2 U/μL DNAse I) and cells were lysed by sonication using a Cup Horn accessory according the manufacturers recommendation (Fisher Scientific Sonic Dismembrator model 550). The lysate was then clarified by two rounds of centrifugation at 18,000g at 4°C. Following solubilization of the pellet portion in SDS-PAGE loading buffer, this and the clarified lysates were analyzed by SDS-PAGE to evaluate relative expression of each uricase, and partitioning of the expressed product between soluble and insoluble material.

The native *Arthrobacter globiformis* uricase sequence contains a single Cys residue which is at the C-terminus of the protein. The C-terminal region of the *Arthrobacter globiformis* uricase sequence is not conserved across >200 aligned uricase sequences and appears to be an extension that may not be necessary for enzymatic activity. Consequently, the *Arthrobacter globiformis* uricase used in the work described here was truncated by 11 amino acids at the C-terminus (HPIWSNIAGFC) such that the protein contained no Cys (S1 Fig).

Uricase proteins were purified from clarified lysates by immobilized metal affinity chromatography using nickel-charged HiTrap Chelating HP columns (GE Healthcare Lifesciences) according to the manufacturer’s protocol. Following purification, samples were dialyzed into PBS, and analyzed by SDS-PAGE, electrospray mass spectrometry, and size exclusion chromatography with multi-angle light scattering analysis to determine purity and covalent as well as noncovalent molecular weights. Concentrations of purified protein samples were determined by absorbance at 280 nm, using extinction coefficients calculated according to the method of Gill and von Hippel [47].

### Differential Scanning Calorimetry

For differential scanning calorimetry analysis, proteins were analyzed at a concentration of 1 mg/mL in PBS buffer using a VP-DSC (MicroCal, LLC). Samples were heated from 30 to 110°C using a scan rate of 60°C/h. The buffer blank was subtracted from all data. The thermograms were further baseline corrected using a progressive baseline and protein concentration normalization.

### Substrate Depletion Assay

Depletion of UA substrate is a common method for assessing uricase activity [48, 49]. In the substrate depletion assay, uricase, UA, and phosphate buffer were incubated separately for 1 hour at the stated temperature (typically 30°C). Uricase was then diluted to 1 μg/mL and combined with different concentration of UA (400 μM diluted down 1:1.6 to 23.8 μM) in 0.1M phosphate buffer (PB), pH 7.4. In some assays (when protein had unconjugated Cys) 1 mM dithiothreitol was added to the assay. The Molecular Devices reader temperature was set to the 30°C. Absorbance measurements at 292 nm were captured every 20 seconds for a period of 10 minutes. The rate of UA degradation (μmol/min) was calculated by SoftMax Pro software.

### M21 Cell Adhesion Assay

To measure integrin-mediated adhesion of uricase variants, 96-well Microlite 2 plates (ThermoScientific) were coated overnight at 4°C with phosphate buffered saline, pH 7.4 (PBS; Life Technologies) or PBS containing 0–100 μg/ml of either human fibronectin (Sigma-Aldrich), Fab9mCys IgG, or uricase proteins. Fab9mCys is a human IgG generated at MedImmune that contains a RGD motif grafted into the CDR H3 loop to confer integrin binding activity [50]. After substrate coating, wells were washed once with PBS and then blocked for 1 hour at room temperature with 1% w/v bovine serum albumin in PBS (Sigma-Aldrich). Human melanoma M21 cells were harvested with Cell Dissociation Buffer (Life Technologies) and resuspended in RPMI medium (Life Technologies) at 5x10^6^ cells/ml. Cells were labeled for 30 min at room temperature with 5 μM calcein-AM (Millipore EMD) and then washed three times with RPMI and once with PBS. 7.5x10^4^ cells/well were plated and allowed to adhere for 60 min at 37°C. After adhesion, wells were washed three times with PBS and cell binding was measured using an Envision Multilabel plate reader (PerkinElmer) at 494 nm absorbance, 517 nm excitation. The experiment was performed in duplicate.

To test blocking of M21 cell adhesion by synthetic cyclic RGD (cRGD; sequence RGDK) or RGE (sequence GRGESP) peptides (American Peptide Company), wells were coated with 5 μg/ml uricase or Fab9mCys IgG. Adhesion of labeled M21 cells was performed in the presence of cRGD or RGE peptides at concentrations from 0–100 μg/ml.

### Uricase Protein Immunogenicity Analysis (Epibase^®^)

Donors were recruited at Phase I clinical trials units in the UK. All samples were collected under an ethical protocol approved by the London—Surrey Borders Research Ethics Committee and written informed consent was obtained from each donor prior to sample donation. All samples were stored according to the terms of Lonza’s Human Tissue Authority license for the use of samples in research. PBMC from healthy donors were prepared from whole blood within six hours of blood withdrawal. Cells were cryopreserved in vapor phase nitrogen until used in the assays. The quality and functionality of each PBMC preparation was analyzed using short term polyclonal T cell activation with anti-CD3, and 7 day activation with positive controls such as KLH to assess naïve T cell responses.

PBMC from selected donors were thawed, washed and seeded onto 96-well plates at a density of 3x105 cells per well. Test proteins and formulation buffers, diluted in assay media, were added to the cells at specified concentrations. Assay media alone was used as a blank and KLH was used as a naïve positive control. PBMCs were incubated for 7 days in a humidified atmosphere at 37°C and 5% CO2. On day 7, PBMCs were labelled for surface phenotypic CD3^+^ and CD4^+^ markers and for DNA-incorporated EdU (5-ethynyl-2’deoxyuridine), used as a cell proliferation marker. The percentage of CD3^+^CD4^+^EdU^+^ proliferating cells was measured using a Guava easyCyte 8HT flow cytometer and analysed using GuavaSoft InCyte software. A preliminary experiment assessed the influence of the reference test proteins (*Arthrobacter globiformis* uricase) and formulation buffers on KLH-induced T cell proliferation. A fixed concentration of KLH (30 μg/ml) was co-cultured with the test proteins at 3 different concentrations (1, 10 and 30 μg/ml). The formulation buffers were tested at a volume equivalent to the 30μg/ml dose of test protein. All subsequent experiments used the optimized conditions (30μg/ml concentration) to assess T cell proliferation responses to the test proteins in 202 healthy donors.

Proliferating CD4^+^ T cells were defined as EdU-A488 positive CD3^+^CD4^+^ cells. S2 Fig outlines the gate setting strategy for the identification of proliferating CD3^+^CD4^+^ T cells. A region gate was used to select healthy single cells on the FSC/SSC plot. The CD3^+^CD4^+^ T cell population was selected using sequential gating on CD3-expressing (CD3^+^) and then on CD4-expressing (CD4^+^) cells. Proliferation of CD3^+^CD4^+^ cells was analyzed by the fluorescence intensity of EdU-A488, a DNA incorporation marker. Using a dot plot of CD4-A647 and EdU-A488 on the gated CD3^+^CD4^+^ cells, proliferating cells were quantified as the number of CD3^+^CD4^+^EdU^+^ cells per well. Donor specific gates were set and analysis was performed. This resulted in a.csv file that contained plate identifications and all GUAVA measurement parameters (number of events, percentages, mean fluorescence intensities). Each test condition was carried out in 8-plicate (n = 8).

Assessment of T cell responses to the test products consisted of the comparison of T-cell proliferation in antigen-treated wells to untreated reference wells. Stimulation indices (SI values) describe the ratio of the number of proliferating CD3^+^CD4^+^ T cells in antigen treated *versus* untreated wells. Statistical analysis of protein induced T-cell proliferation at the individual donor level is driven by the experiment design, with all protein treated and control wells on the same plate. To minimize contributions of experiment design, randomized plate layouts were applied. At the individual donor level the immune response to each test protein was evaluated by comparing to the blank or buffer condition, indicating if each donor elicited a significant response. Therefore estimates of the average number of activated or proliferating cells from each condition were obtained from a linear model (using the statistical software R). Subsequently contrasts (SI) between the estimated averages were calculated. For each donor/protein combination, SI-values, 95% confidence intervals and p-values for the hypothesis SI = 1, were calculated. Here, an antigen inducing an immune response is defined when the SI is ≥2. The SI threshold is pre-determined from in-house data using positive and negative control proteins. The SI ≥2 should also be supported by a p-value < 0.05 to be identified as a positive response.

### Site Specific PEGylation

PEGylation reactions were performed in sodium phosphate buffer, pH 7.0. Maleimide-functionalized PEG-10 (10kDa, Sunbright MA-100) was obtained from NOF America Corporation (White Plains, NY). PEG was added to the reaction at 1000 μM and incubated for 3 hours. PEGylation reactions were quenched after selected time points by the addition of dithiothreitol to a final concentration of 10 mM and analyzed by analytical reverse-phase high performance liquid chromatography using an YMC-Pack Protein-RP column (250x2.0 mm, S-5μm) from YMC America (Allentown, PA, USA) with an Agilent HPLC1200 system. Mobile phase A was 0.1% TFA in water and mobile phase B consisted of 0.1% TFA in acetonitrile. The sample was eluted with a linear gradient of increasing mobile phase B at a flow rate of 0.4 mL/min. Elution profiles were monitored by UV absorbance at 280 nm.

### Human Serum Assay

Di-PEGylated uricase activity was evaluated in 50% human serum (Bioreclamation) at 37°C. The assay was performed as follows: UA, phosphate buffer, and serum were warmed to 37°C. All reaction steps were done at 37°C. Uricase was diluted to 8 μg/mL in serum for 20–30 minutes to deplete endogenous UA in the human serum sample. An equal volume titrated UA in phosphate buffer was added and the reaction was stopped at 0, 1, 2, 4 or 6 minutes using 50% perchloric acid. Perchloric acid has been shown to precipitate protein but does not precipitate the UA [38, 39]. The precipitate was pelleted and 100 μL of the supernatant was transferred to a UV plate. Absorbance was measured at 292 nm. Rate was calculated by plotting the slope of the 4 time-points at each UA concentration using SoftMax Pro software.

Human serum based stability assay: Di-PEGylated uricase was incubated in 50% human serum for 0, 0.5, 1, 4, or 24 hours at 37°C and then assayed for activity as described above.

### In vivo Rat and Canine PK Studies

Rat PK studies were done with the approval and under the direction of the MedImmune’s Institutional Animal Care and Use Committee (MI-11-0010-002). Wild type 9 week old Sprague Dawley rats were purchased from Harlan. All animals were observed at least once per day. Observations included a detailed checklist of general behavior and appearance. No animals became sick or died during the duration of the study. Body weight of each animal was recorded prior to the study and on the day of termination. Within the protocol, any rats experiencing signs or symptoms of distress or pain were to be euthanized by carbon dioxide.

Two PEGylated uricases (di-PEG T11C, N33C and tri-PEG T11C, N33C, and S142C) were evaluated in a rat study. 4 rats in each group were dosed IV at 5 mg/kg and 10 samples were collected (Day -1), 0.5, 2, 4, 8, 24, 48, 72, 96 and 144 hours post injection per rat. Whole blood was collected in serum separator tubes and frozen. Serum samples were diluted into phosphate buffer and combined with 400 μM UA. Activity analysis was performed as described above at 30°C using the reader setup as in the buffer assay. Standard curves were prepared in diluted serum to match the samples.

Di-PEGylated uricase (T11C, N33C) was evaluated in a canine PK study. All canine studies were perfomed by Covance Laboratories which is fully accredited by the Association for Assessment and Accreditation of Laboratory Animal Care. All procedures in the protocol were approved by their local Institutional Animal Care and Use Committee. 8 male beagle dogs were received from Covance Researfch Products in Cumberland, VA and were acclimated for 1 week. All dogs were 8–9 months of age. Twice daily cageside observations were made for each animal and abnormal findings were recorded (mortality, abnormalities, and signs of pain or distress). Once daily clinical examinations were performed during dosing phase. Food consumption, irritation scoring, body weights, and animal disposition were all included in the protocol procedure. No animals became ill during the study and following the final collection all animals were returned to stock.

3 canines per group were dosed SC at 3 mg/kg or 10 mg/kg and and 2 canines received a SC injection of PBS at an equivalent volume. Whole blood was collected in serum separator tubes and frozen (Day -1), 0.5, 6, 24 hours postdose and on study days 3, 5, 7, 21, 35, 42, and 56. No adverse events were observed during this study. Serum samples were diluted into phosphate buffer and combined with 400 μM UA. Activity analysis was performed as described above at 30°C using the reader setup as in the buffer assay. Standard curves were prepared in diluted serum to match the samples.

Blood UA levels were measured in the following way: Whole blood was immediately crashed with 4 volumes of 10% (w/v) trichloroacetic acid in water to halt all uricase activity. An aliquot of the supernatant was analyzed for quantitative determination of uric acid. An internal standard ([1,3-^15^N_2_]uric acid) was added, and the samples were diluted with methanol. The supernatants were completely dried down and reconstituted with injection solvent (0.1% ammonium hydroxide solution in water) and analyzed by high-performance liquid chromatography with tandem mass spectrometry (LC/MS/MS). An API 5000 triple quadrupole mass spectrometer (AB Sciex), operated in negative TurboIonSpray^®^ mode, was used to monitor the precursor → product ion transitions of *m/z* 167 → 124 and *m/z* 169 → 125 for uric acid and [1,3-^15^N_2_]uric acid, respectively.

## Supporting Information

S1 Table Atomic distances between pairs of Cα atoms across the tetrameric structure. (TIF)

S1 Fig Amino acid sequences 8 uricases. (TIF)

S2 Fig Gate setting strategy for the identification of proliferating CD3^+^CD4^+^ T-cells. (TIF)

S3 Fig Peptide blocking assay using soluble control RGD or RGE. (TIF)

S4 Fig HLA-DRB1 frequencies in the Caucasian population relative to the study population. (TIF)

S5 Fig Cys-containing and PEGylated constructs activity assay curves. (TIF)

S1 Data Fig 3 data Prism file. (PZF)

S2 Data Fig 6A data LONZA Epibase data file. (PZF)

S3 Data Fig 6B LONZA Epibase data file. (PZF)
