# Supplementary figures and images for: A Therapeutic Uricase with Reduced Immunogenicity Risk and Improved Development Properties
Source: PLoS One. 2016 Dec 21;11(12):e0167935. doi: 10.1371/journal.pone.0167935 (PMC5176304; doi:10.1371/journal.pone.0167935)

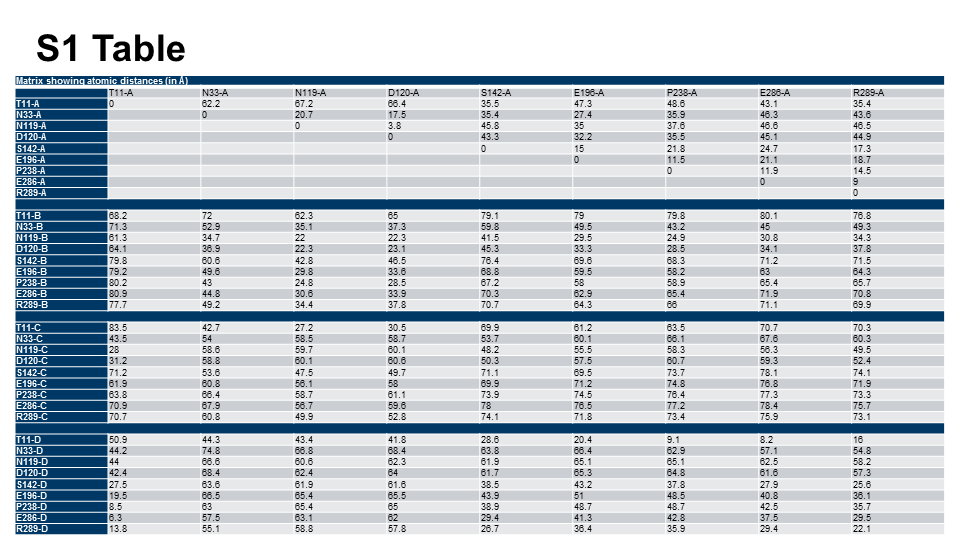

Supplement: S1 Table — (TIF) [file pone.0167935.s001.tif]

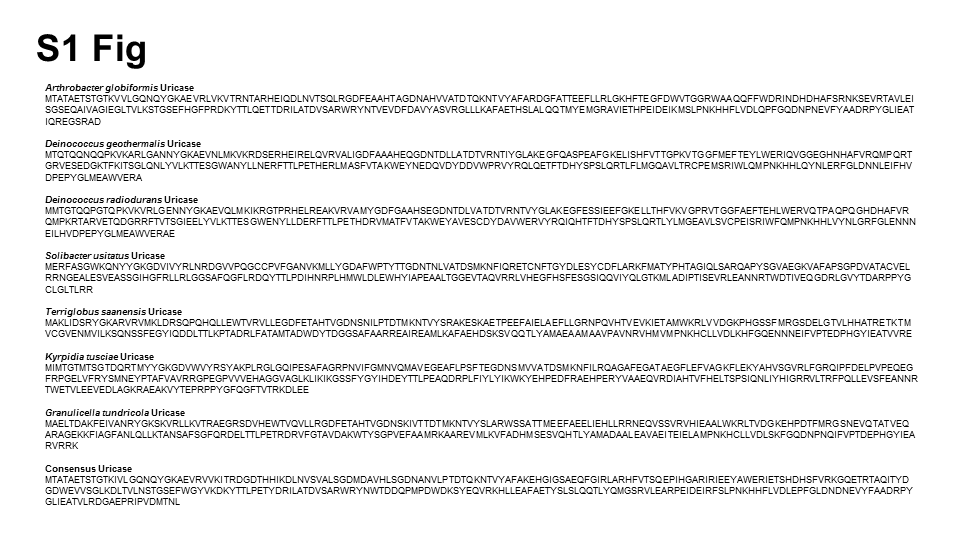

Supplement: S1 Fig — (TIF) [file pone.0167935.s002.tif]

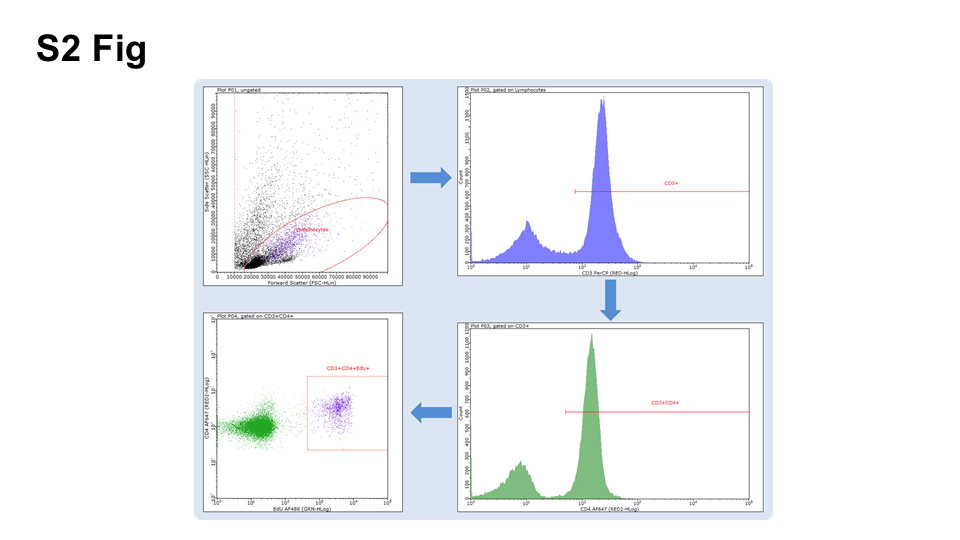

Supplement: S2 Fig — (TIF) [file pone.0167935.s003.tif]

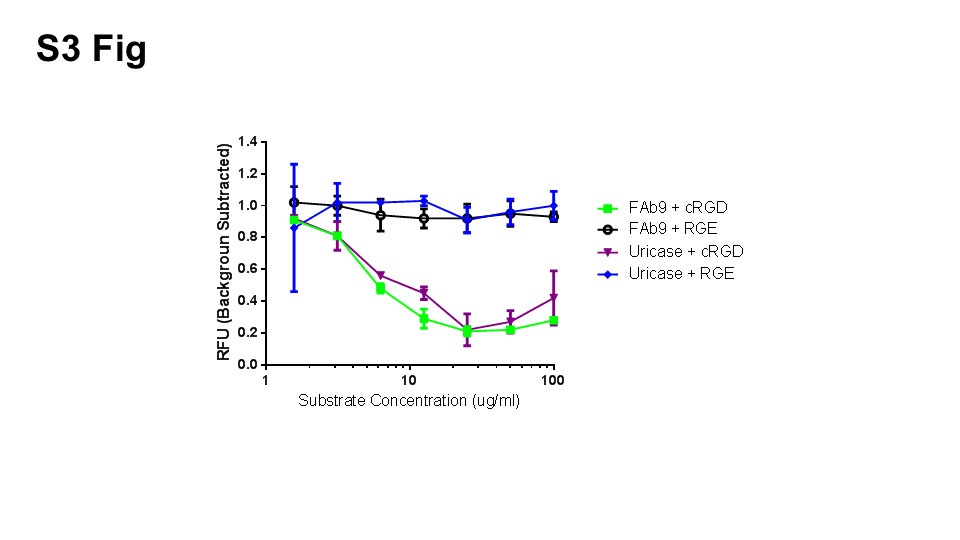

Supplement: S3 Fig — (TIF) [file pone.0167935.s004.tif]

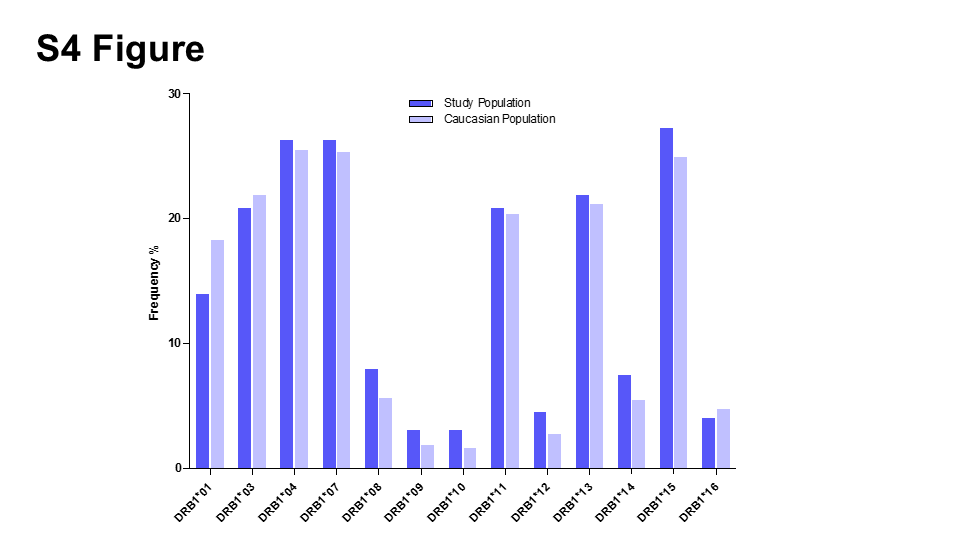

Supplement: S4 Fig — (TIF) [file pone.0167935.s005.tif]

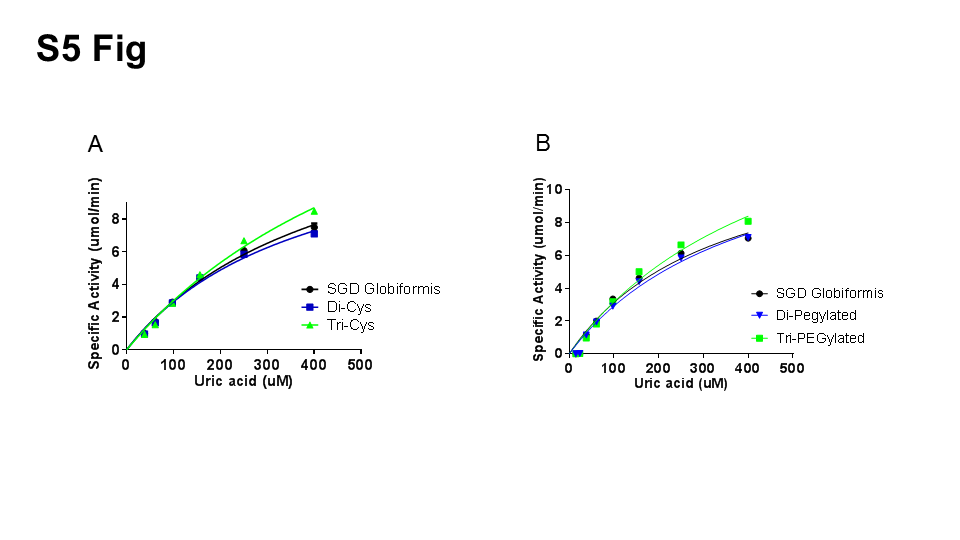

Supplement: S5 Fig — (TIF) [file pone.0167935.s006.tif]
